# Allometric Growth of Feeding and Locomotor Structures During Early Ontogeny of Rabbitfish (*Siganus fuscescens*)

**DOI:** 10.3390/ani16050777

**Published:** 2026-03-02

**Authors:** Lynn Nuruki, Aki Miyashima, Yasuo Agawa, Yoshifumi Sawada

**Affiliations:** 1 Graduate School of Agriculture, Kindai University, Nakamachi 3327-204, Nara 631-8505, Nara, Japan; 953962@kindai.ac.jp; 2 Aquaculture Research Institute, Kindai University, Oshima 1790-4, Kushimoto 649-3633, Wakayama, Japan; aki.miyashima@itp.kindai.ac.jp (A.M.); agawa@kindai.ac.jp (Y.A.)

**Keywords:** mottled spinefoot, larvae, ontogeny, allometry, aquaculture, early development, flexion, feeding

## Abstract

Early survival of marine fish larvae depends on the timely development of feeding and swimming functions. This study examined ontogenetic changes in relative growth patterns of feeding- and locomotion-related body parts in the mottled spinefoot rabbitfish, *S. fuscescens*. Larvae and early juveniles were reared under controlled conditions, and morphometric measurements were analyzed using log–log segmented regression. Body length increased gradually during the early larval stage and accelerated after approximately 10 days post-hatching. Three developmental phases were identified, with breakpoints at approximately 5 mm, 7–9 mm, and 17–19 mm body length. In the early larval phase (NL < ~5 mm), eye diameter, upper jaw length, snout length, and caudal peduncle depth showed strong positive allometry, indicating rapid acquisition of feeding and swimming functions. This was followed by a mid-larval phase characterized by near-isometric growth and stabilized body proportions. During the late larval to early juvenile phase, body depth and caudal peduncle depth again exhibited positive allometry, reflecting reorganization toward juvenile morphology. These results reveal a stage-specific growth strategy in *S. fuscescens* and provide a morphological basis for improving larval rearing and feeding practices.

## Simple Summary

The early survival of marine fish larvae depends strongly on how quickly they acquire basic abilities such as vision, feeding, and swimming. In this study, we examined how different body parts develop during early growth in the mottled spinefoot rabbitfish (*Siganus fuscescens*), a coastal herbivorous fish important to both ecosystems and aquaculture. Our results show that rabbitfish larvae do not grow evenly across all body parts. Instead, structures involved in feeding and movement tend to develop earlier than overall body size, which likely helps larvae begin feeding successfully soon after hatching. As development proceeds, body proportions become more balanced, and later change again as fish prepare for juvenile life in shallow coastal habitats. This developmental pattern differs from that reported for several carnivorous marine fishes and reflects a growth strategy adapted to the rabbitfish’s herbivorous lifestyle. Understanding these growth characteristics provides useful biological insight for improving larval rearing practices and for better understanding how fish larvae adapt to their environments.

## 1. Introduction

Rabbitfishes of the genus *Siganus* are widely distributed across tropical and sub-tropical Indo-Pacific coastal regions and represent an ecologically and economically important group of herbivorous fishes. Among them, the mottled spinefoot *S. fuscescens* is common throughout the western Pacific, Southeast Asia, and the Ryukyu–Japanese archipelago [1]. Recent genetic studies have revealed complex phylogeographic structures and partially isolated regional populations [2,3,4], suggesting that local adaptation and region-specific reproductive characteristics may occur across its broad distribution. In many Indo-Pacific countries, rabbitfishes constitute important coastal fisheries resources and are widely consumed [5]. Their herbivorous diet, relatively fast growth, and favorable consumer acceptance have further contributed to increasing interest in their aquaculture potential [5].

Recent studies have further clarified the reproductive biology and spawning seasonality of the mottled spinefoot rabbitfish *S. fuscescens*, highlighting regional variation and environmental influences on reproductive timing [6].

Despite this long-standing interest, the successful seed production of *Siganus* species remains challenging, particularly during the early larval stages. Studies on *S. guttatus* have provided detailed information on spawning characteristics, yolk absorption, timing of mouth opening, and the narrow size range of prey that larvae can ingest at first feeding, all of which contribute to high mortality when appropriately sized prey are not supplied [7,8]. Similarly, the developmental sequence of *S. fuscescens* larvae, including fin formation, pigmentation, and digestive tract extension, has been described [9]. These studies collectively demonstrate that siganid fish larvae exhibit rapid yolk depletion, very small initial mouth size, and a strict requirement for suitably sized prey immediately after first feeding. Although these findings are derived from closely related *Siganus* species, such comparisons are justified primarily by their shared phylogenetic background and broadly similar early-life feeding modes as herbivorous or omnivorous fishes. These species exhibit common early developmental constraints, including rapid yolk depletion, small initial mouth size, and strong dependence on visual feeding cues immediately after first feeding. Nevertheless, potential species-specific eco-functional differences cannot be excluded, and the present comparisons are intended to provide developmental context rather than to imply direct equivalence across species. For *S. fuscescens*, inadequate feeding during the onset of exogenous feeding—particularly between 2 and 6 days post-hatching—has been identified as a major cause of poor survival in fingerling production [7]. Specifically, the relative growth of feeding-related structures such as the eye, snout, and jaw provides morphological predictors of the prey sizes that larvae can effectively capture and ingest at different developmental stages. These allometric indicators also define practical thresholds at which larvae are likely to tolerate changes in prey type, such as transitions from small rotifers to larger live feeds. Therefore, quantifying relative growth patterns offers a morphological basis for optimizing feeding schedules and rearing practices in hatchery production.

Early feeding success in marine fish larvae is influenced by both environmental conditions and intrinsic developmental constraints, particularly the development of feeding-related external morphology and the digestive system. While many marine larvae are visual feeders whose feeding performance is strongly affected by light intensity, successful prey ingestion and assimilation during the first-feeding period ultimately depend on whether feeding-related structures and digestive capacity are sufficiently developed. In rabbitfishes, recent hatchery observations indicate that *S. fuscescens* larvae exhibit strong positive phototaxis immediately after the onset of exogenous feeding, which under artificial light environments can induce maladaptive behaviors such as wall aggregation and surface-pecking, potentially increasing energy expenditure and early mortality.

Although previous studies have described aspects of reproductive biology, external morphology, and early feeding in *Siganus* species [7,8,9,10], important gaps remain in our understanding of early morphological and functional development in *S. fuscescens*. Kitajima et al. [9] reported the growth relationships of several external traits, including body depth, preanal length, eye diameter, second dorsal spine length, pelvic spine length, and the allometric relationship between total length and body weight. However, feeding-related structures such as snout length, upper jaw length, and head length were not examined, and their ontogenetic growth patterns remain unknown. Kitajima et al. [9] also provided a description of general gut morphology, and the present study re-visits these observations to confirm and extend their findings. However, beyond documenting size relationships of individual traits, previous studies did not establish a quantitative framework for how coordinated allometric changes in feeding- and sensory-related structures define functional developmental phases. In particular, the timing and magnitude of growth transitions that mark shifts in feeding capacity and locomotor performance have not been identified in *S. fuscescens*. The present study addresses this gap by applying segmented allometric analysis to detect ontogenetic breakpoints that delineate functionally distinct growth phases.

Further information on early development has been provided by studies on other *Siganus* species. Juario et al. [7] documented the spawning characteristics, yolk absorption, timing of mouth opening, and initial feeding performance of *S. guttatus*. Bagarinao [8] emphasized the narrow timing of the first-feeding window and the critical importance of providing appropriately sized prey. However, these studies focused primarily on general larval morphology and feeding onset and did not provide detailed analyses of digestive tract differentiation or the relative growth of feeding-related structures in *S. fuscescens*. In *S. fuscescens*, Kitajima et al. [10] noted that eye enlargement and increases in body depth accompany the onset of exogenous feeding, indicating that early somatic growth is aligned with the development of visual and oral structures that facilitate prey detection and ingestion. These observations highlight the importance of feeding-related morphology during the transition to external feeding. However, key traits such as head length, snout length, and upper jaw length were not quantified in their study, and the ontogenetic growth patterns of these structures remain insufficiently understood.

In *S. fuscescens*, detailed information on the ontogeny of digestive tract development and the allometric growth of feeding-related structures remains scarce, particularly during the earliest post-hatching stages. Although Kitajima et al. [9] provided valuable descriptions of the external morphology of the digestive tract, their observations focused mainly on larvae from 14 days post-hatching onward, leaving the formative period of digestive and feeding-related structures largely undocumented. Such early-stage information is essential for designing feeding strategies that match larval developmental capacity and for improving hatchery protocols. These observations suggest that early mortality in *S. fuscescens* larvae may arise not from delayed morphological development per se, but from mismatches between larval functional capacity and hatchery feeding and environmental conditions.

The present study therefore aims to clarify the early ontogeny of *S. fuscescens* by (1) describing basic growth patterns and overall morphological development from hatching onward, (2) quantifying the relative growth of feeding-related structures, and (3) interpreting the development of feeding-related external morphology in the context of digestive system maturation based on previous studies. By identifying developmental milestones associated with prey size selectivity and inferred digestive capacity, this study provides foundational biological information for guiding first-feeding strategies and improving the reliability of fingerling production in this ecologically and economically important species.

## 2. Materials and Methods

### 2.1. Test Larvae and Juveniles and Their Rearing Methods

A total of 8000 fertilized eggs obtained from 14 broodstock individuals maintained at the Oshima Experiment Station were randomly distributed into five identical indoor 1-t cylindrical tanks at hatching. Multiple tanks were used to ensure standard hatchery conditions and to minimize potential tank-specific effects, while all tanks were maintained under identical and controlled environmental conditions throughout the rearing period.

Seawater was supplied continuously at a flow rate of 0.4 L min^−1^ using an overflow system. Dissolved oxygen saturation ranged from 81.2% to 107.3%, salinity was maintained at 32.0 ± 1.8 PSU, and water temperature ranged from 25.3 °C to 31.0 °C. Light intensity was set at 16,000 lx, and the photoperiod was maintained at 14 h light and 10 h dark (14L:10D; light phase from 07:30 to 21:30). These conditions were identical among all tanks.

Water temperature, dissolved oxygen (DO), salinity, and pH were recorded twice daily (AM and PM) throughout the pre-transfer period (0–15 dph). To evaluate potential differences in water-quality conditions among tanks during this period, linear models were fitted for each parameter with days post-hatching (dph) and time of day (AM/PM) as covariates and tank as a factor. Because larvae were transferred and pooled among tanks after 16 dph, statistical comparisons among tanks were restricted to the pre-transfer period.

From 2 to 30 days post-hatching (dph), larvae were fed rotifers at a density of 10–30 individuals mL^−1^. Rotifers were enriched with freshwater Chlorella fortified with eicosapentaenoic acid (EPA) and docosahexaenoic acid (DHA). *Artemia* nauplii were supplied from 15 to 40 dph, starting at approximately 10 individuals per larva and gradually increasing according to larval growth.

From 22 dph onward, larvae were additionally fed a formulated commercial diet, and fresh vegetables were introduced from 25 dph. During the rotifer and *Artemia* feeding phases (2–21 dph), larvae were fed once daily in the morning. After 22 dph, feeding frequency and amounts were adjusted to apparent satiation based on observations of larval feeding behavior and swimming activity (e.g., changes in swimming speed and prey capture frequency), which are commonly used as practical indicators of feeding status in aquaculture studies of marine fish larvae (e.g., [10,11]).The seawater used for rearing was treated by ultraviolet sterilization and filtration. When necessary, concentrated *Chlorella* (Fresh Chlorella V-12; Chlorella Industry Co., Ltd., Tokyo, Japan) was added at appropriate concentrations.

### 2.2. Sampling and Measurement of Larvae and Juveniles

At each age (0–40 dph), 5–30 individuals were randomly collected as the daily sample. When multiple tanks were available, individuals were taken from more than one tank to avoid tank-specific bias. Larvae collected from different tanks at the same sampling age were pooled at the time of sampling, and tank identity was therefore not retained for individual specimens. This sampling design was adopted to obtain representative individuals under identical rearing conditions and to minimize potential bias associated with specific tanks.

All individuals collected at the same age were pooled and treated as a single sample. The sampled fish were anesthetized with eugenol (FA100; Bussan Animal Health Co., Ltd., Osaka, Japan) at a concentration of 500 ppm and then euthanized in ice water. Digital images were subsequently captured using a stereomicroscope system (cellSense standard version 1.16; OLYMPUS Corp., Tokyo, Japan; SZX7; OLYMPUS Corp., Tokyo, Japan), and morphometric measurements were obtained from the images. In total, 358 individuals spanning a body-length range of 2.07–57.87 mm were included in the quantitative analyses. This dataset covers all major larval and early juvenile stages examined in this study. Developmental stages of rabbitfish were classified according to Kendall (1984) [12].

For the assessment of absolute growth, notochord length (NL) and standard length (SL) were measured and collectively defined as body length (BL). For relative growth analysis, the following morphological traits were measured: total length (TL), body length (BL), preanal length (PAL), head length (HL), snout length (SnL), body height (BH), head height (HH), caudal peduncle depth (CPD), upper jaw length (UJL), and eye diameter (ED).

Because the primary objective of this study was to characterize individual-level allometric growth trajectories rather than to compare rearing conditions among tanks, individuals collected from different tanks were pooled for the regression-based analyses. Preliminary inspection, based on visual comparison of size-frequency distributions and summary statistics (mean ± SD) of body length and major morphometric traits among tanks at overlapping sampling days, did not reveal systematic differences among tanks; therefore, tank effects were not explicitly modeled. Nevertheless, we acknowledge that individuals reared in the same tank may not be fully independent due to shared environmental conditions, and this limitation is considered when interpreting the results.

### 2.3. Statistical Analysis

#### 2.3.1. Software and Analytical Environment

Statistical analyses were performed using R software (version 4.3.1). Additional data checking and basic statistical summaries were conducted using EZR (Easy R, version 1.63), which is widely used in biological and medical research. EZR was used primarily for data inspection and descriptive statistics, whereas all regression and segmented analyses were conducted directly in R. Segmented regression analyses were performed using the segmented package in R [13]. Data organization and calculation of basic descriptive statistics were conducted using Microsoft Excel as a supplementary tool.

#### 2.3.2. Data Preprocessing and Outlier Detection

Morphological measurements, including pre-anal length, snout length, head length, head height, caudal peduncle depth, body height, total length, eye diameter, and upper jaw length, were recorded for each individual at each sampling age.

Prior to statistical analysis, outliers were identified using the interquartile range (IQR) method to minimize the influence of measurement errors and extreme values. Specifically,

- the first (Q1) and third quartiles (Q3) were calculated,
- the interquartile range was defined as IQR = Q3 − Q1, and
- values below Q1 − 1.5 × IQR or above Q3 + 1.5 × IQR were identified as potential outliers.

Outliers identified by the IQR method were carefully inspected on a case-by-case basis, and values were excluded only when they were considered likely to result from measurement or recording errors rather than representing biologically meaningful variation.

#### 2.3.3. Allometric Growth Analysis (Log–Log Regression)

Allometric relationships between each morphological trait (*Y*) and the reference body length (X) were analyzed using the allometric equation:

*Y* = *aX*^*b*^

After logarithmic transformation, the equation was expressed as:

ln(*Y*) = ln(*a*) + *b* ln (*X*) (1)

Natural logarithms (ln) were used for all transformations. All measurements were positive values, and no zero values were present in the dataset; therefore, logarithmic transformation was applied without additional correction.

Linear regression analyses were then performed on the log-transformed data.

The regression coefficient b was interpreted as the allometric growth coefficient, where

- *b* = 1 indicates isometric growth,
- *b* > 1 indicates positive allometry, and
- *b* < 1 indicates negative allometry.

The allometric coefficient *b* was statistically evaluated by testing whether it differed significantly from isometric growth (*b* = 1) using *t*-tests.

Notochord length was used as the reference body length prior to notochord flexion, whereas standard length was used thereafter, reflecting ontogenetic changes in body structure.

#### 2.3.4. Segmented Regression Analysis and Inflection Point Estimation

Segmented regression models allowing up to two breakpoints (i.e., up to three linear segments) were fitted to the log–log relationships between body length and each morphometric trait. Breakpoints were identified using a model-selection approach rather than visual inspection, by comparing competing segmented regression models with different numbers of breakpoints based on Akaike’s Information Criterion. To detect body length thresholds at which allometric growth patterns changed during development, segmented regression analysis was applied to the log–log transformed data.

Initially, a simple linear regression model was fitted to each trait. Subsequently, segmented regression models were constructed using the segmented package in R to allow the regression line to be divided into multiple segments with different slopes [14].

Segmented regression models with zero to two breakpoints (corresponding to one to three linear segments) were fitted to the log–log relationships between body length and each morphometric trait. Candidate models were compared by sequentially increasing model complexity, and the optimal number of breakpoints was selected based on Akaike’s Information Criterion (AIC; [15]), parameter stability, residual diagnostics, and biological interpretability of the estimated breakpoints.

The general form of a segmented regression model with two breakpoints is given by:

$$ln(Y)=\{\begin{array}{cc} \alpha_{1}+b_{1}ln(X), & \left(X<\psi_{1}\right)\\ \alpha_{2}+b_{2}ln(X), & \left(\psi_{1}\leq X<\psi_{2}\right)\\ \alpha_{3}+b_{3}ln(X), & \left(X\geq \psi_{2}\right) \end{array}$$

where $\psi_{1}$ and $\psi_{2}$ denote the estimated breakpoints, and $b_{1}$, $b_{2}$, and $b_{3}$ are the segment-specific allometric coefficients. Breakpoints were estimated on the logarithmic scale and back-transformed to millimeters for biological interpretation. Standard errors of breakpoint estimates were calculated to quantify estimation uncertainty. The breakpoints and segment-specific slopes reported in Table 1 correspond to the final selected model for each trait; traits supported by fewer breakpoints are represented by correspondingly simpler models. For total length, the two-breakpoint model was strongly supported over simpler alternatives (ΔAIC = 79.7 relative to the one-breakpoint model). Among candidate models, the final model was selected primarily based on the lowest AIC, provided that estimated breakpoints were biologically interpretable and associated with stable slope estimates.

#### 2.3.5. Model Evaluation and Interpretation

For each regression model, regression coefficients, standard errors, coefficients of determination (*R*^2^), and the number of estimated breakpoints were evaluated.

When multiple breakpoints were detected, their relevance was assessed in relation to ontogenetic stage transitions, rather than relying solely on statistical criteria.

#### 2.3.6. Statistical Significance

Statistical significance was assessed at a 5% significance level (*p* < 0.05).

Differences in regression slopes and improvements in model fit were evaluated based on *t*-tests and the estimated parameters of the regression models.

## 3. Results

### 3.1. Environmental Conditions During the Rearing Period

During the pre-transfer period (0–15 dph), no significant differences among tanks were detected for any water-quality parameter, including water temperature, dissolved oxygen, salinity, and pH (Table S1). Salinity and pH varied significantly with days post-hatching, and salinity also differed between AM and PM measurements; however, these temporal variations were consistent across tanks.

### 3.2. Absolute Growth of Notochord Length or Standard Length

**Figure 1:**
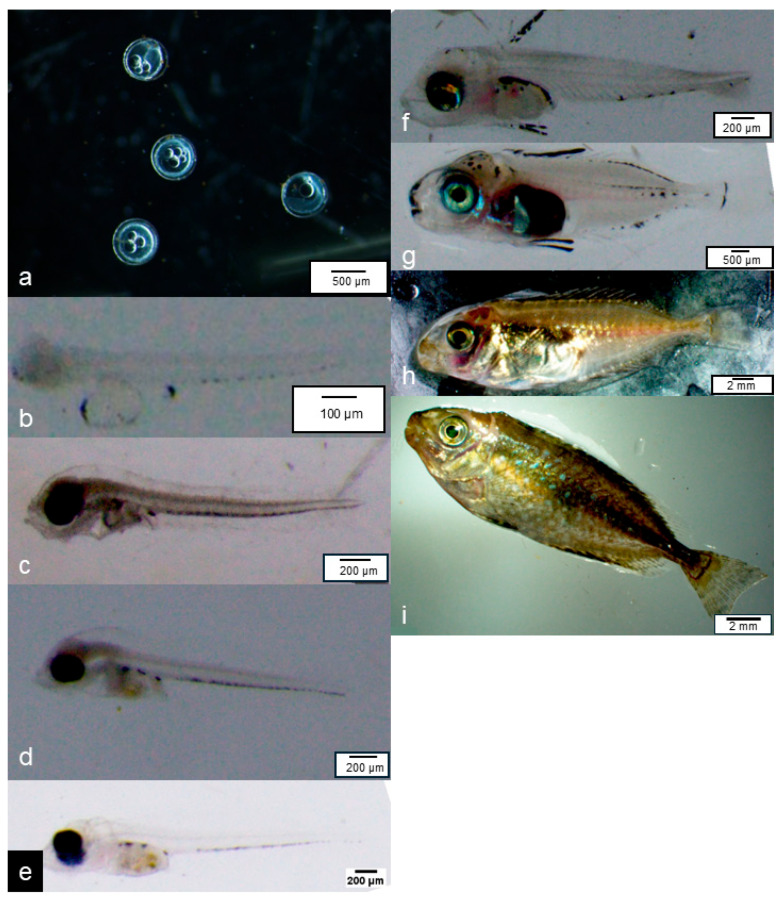
Representative images showing the developmental stages of Mottled spinefoot (*S. fuscescens*); (**a**): Fertilized egg, (**b**): 0 days post-hatch (dph), early larval stage, (**c**): 2 dph, early larval stage, (**d**): 3 dph, early larval stage, (**e**): 7 dph, flexion-stage larva, (**f**): 11 dph, flexion-stage larva, (**g**): 19 dph, late flexion-stage larva, (**h**): 34 dph, juvenile, (**i**): 40 dph, juvenile.

After sampling, the experimental fish were photographed using a microscope-mounted camera. From the obtained images, individuals and ages that were considered representative of each developmental stage were selected and are presented in Figure 1.

**Figure 2:**
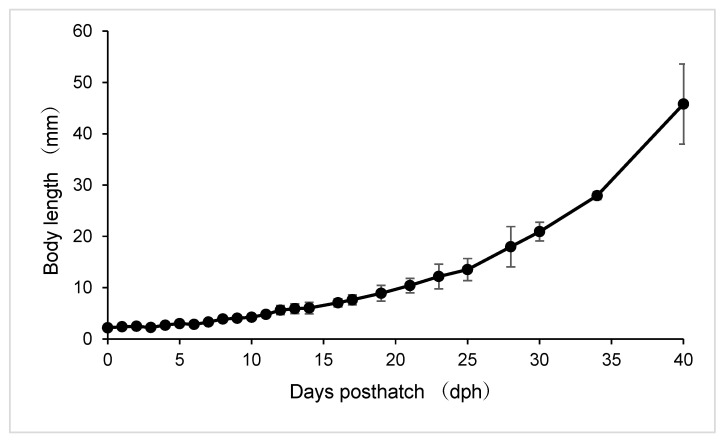
Absolute growth of body length (notochord length or standard length) of mottled spinefoot larvae and juveniles. Vertical lines indicate standard deviations (*n* = 30).

Changes in body length during early development are shown in Figure 2. Mean notochord length (NL) increased gradually from hatching to approximately 10 dph, remaining below 5 mm during this period. At hatching and during the yolk-sac stage (0–2 dph), NL ranged from approximately 2.1 to 2.3 mm. From 3 to 10 dph, NL increased steadily to approximately 4–5 mm, after which growth accelerated, reaching approximately 10–12 mm by 20 dph.

After 10 dph, growth in body length accelerated markedly. NL increased from approximately 5 mm at 10 dph to about 10–12 mm by 20 dph. Following the transition to the juvenile stage, standard length (SL) increased rapidly, reaching approximately 20 mm by 30 dph, approximately 28 mm by 35 dph, and exceeding 40 mm by 40 dph. Variability in body length increased with age, particularly after 25 dph, as indicated by larger standard deviations. Variability in body length increased with age, particularly after 25 dph, as indicated by larger standard deviations, although variability was not quantified separately within each ontogenetic phase.

### 3.3. Developmental Stage Composition During Larval and Early Juvenile Development

**Figure 3:**
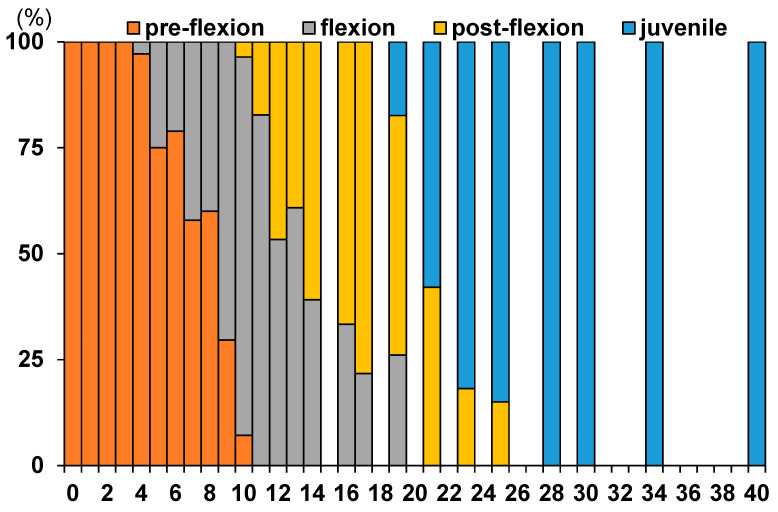
Temporal changes in the relative composition of developmental stages in *S. fuscescens* from hatching to the juvenile stage. Stacked bar charts show the percentage (%) of individuals classified into four developmental stages—preflexion, flexion, postflexion, and juvenile—at each sampling day post-hatch (dph).

The relative proportions of developmental stages (pre-flexion, flexion, post-flexion, and juvenile) changed markedly with days post-hatch (Figure 3). From 0 to 4 dph, all individuals were classified as pre-flexion larvae, accounting for 100% of the population. Flexion-stage larvae first appeared at 5 dph, coinciding with the onset of rapid morphological change.

Between 6 and 9 dph, the proportion of flexion larvae increased rapidly, while pre-flexion larvae declined to less than 10% by 9 dph. Post-flexion larvae began to appear at approximately 10 dph and increased steadily in proportion through 15 dph, during which the population was dominated by flexion and post-flexion stages. From 16 to 19 dph, post-flexion larvae accounted for more than 70% of individuals. Juveniles first appeared at 20 dph and rapidly became dominant, accounting for all individuals from 25 dph onward.

### 3.4. Relative Growth of Body Proportions During Larval and Early Juvenile Development

**Figure 4:**
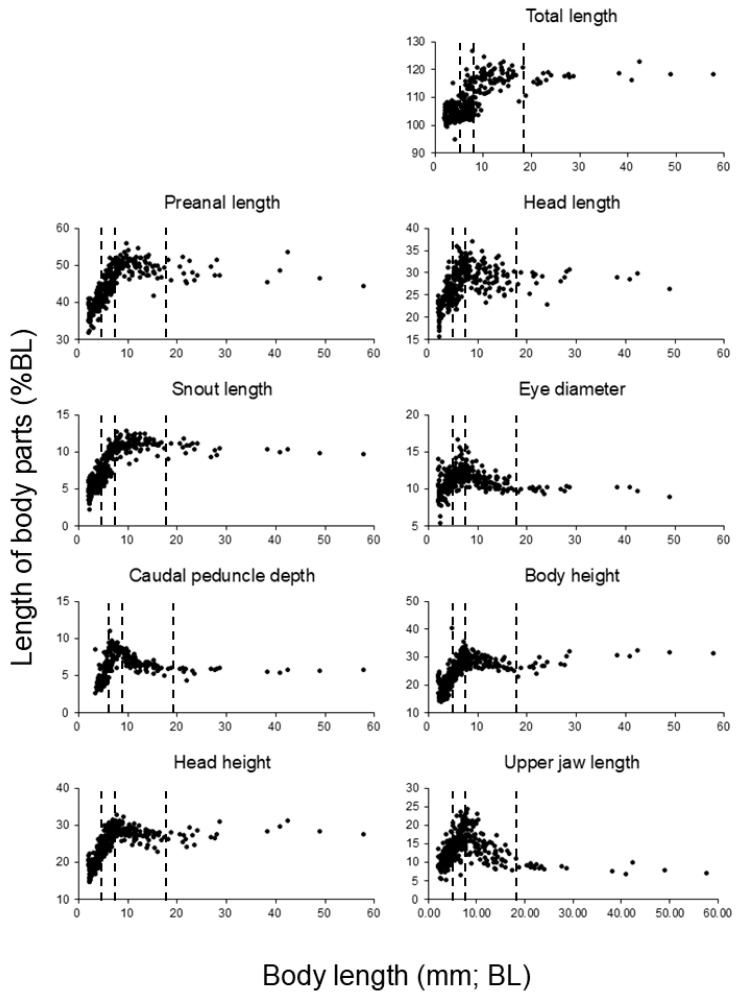
Relative growth of mottled spinefoot (*Siganus fuscescens*) larval and juvenile body parts expressed as a percentage of body length. The *x*-axis represents body length (notochord length before notochord flexion and standard length thereafter, mm), and the *y*-axis represents the relative size of each morphological trait as a percentage of body length (%). Dotted lines indicate the breakpoints in the logarithmic regression.

Relative growth of body parts in *S. fuscescens* was analyzed by expressing each measurement as a percentage of notochord length (NL) during the larval stage and standard length (SL) during the early juvenile stage (Figure 4).

At hatching, larvae were slender with low relative body depth. During the early larval stage (approximately 3–8 dph), body depth and head depth increased rapidly relative to notochord length. Thereafter, these proportions stabilized as overall body length continued to increase. From 8 to 20 dph, NL increased substantially from approximately 4.3 mm to 11.6 mm, whereas body depth and head depth ratios remained relatively stable. This resulted in a gradual elongation of overall body shape during the later larval stage.

Relative growth patterns of feeding-related organs showed distinct ontogenetic trends. Feeding-related traits, including upper jaw length, head length, and eye diameter, were relatively large from the onset of exogenous feeding and changed only gradually during subsequent larval development.

In contrast, relative trunk length increased primarily during later development. From approximately 8 dph onward (NL > 4.3 mm), trunk length increased from approximately 45–48% of NL to 50–55% of NL by 20 dph. The relative length of the caudal region also increased steadily toward the early juvenile stage.

### 3.5. Log–Log Relative Growth and Detection of Developmental Breakpoints

It should be noted that no formal statistical tests were applied to the descriptive results presented in this section; these results are intended to provide an overview of developmental trends, while statistical evaluations are presented in the subsequent analyses. In the log–log relationship between total length and body length, the model with two breakpoints provided the best fit (AIC = −1377.6), indicating two distinct shifts in the allometric scaling pattern. Natural logarithms of notochord length (NL) or standard length (SL) and individual body-part measurements were analyzed using segmented regression to detect breakpoints in relative growth trajectories.

**Figure 5:**
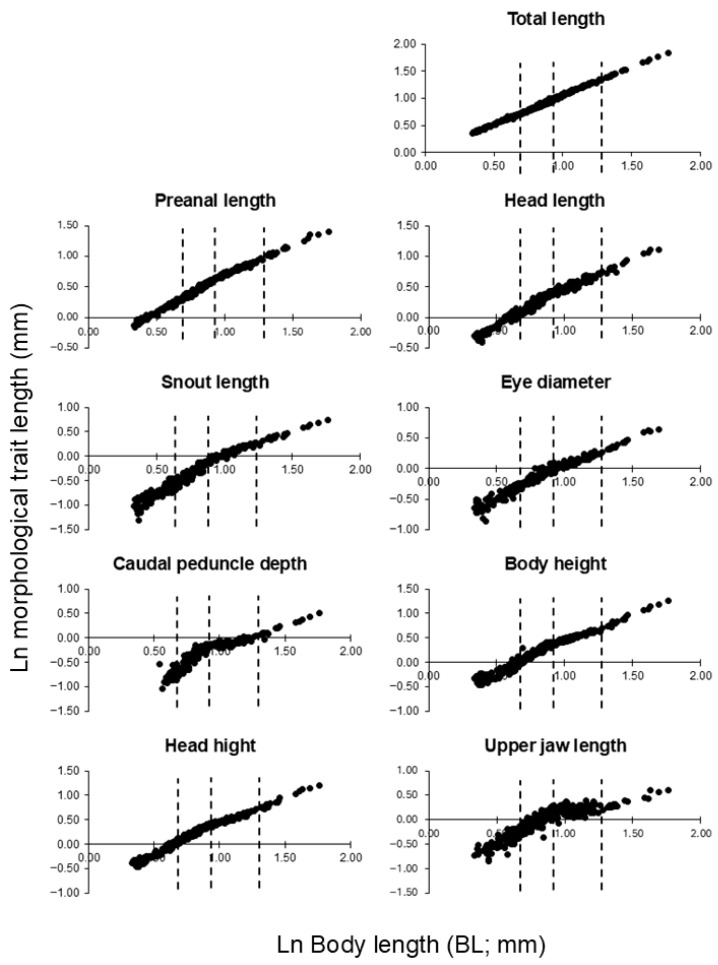
Log–log relationships between body length and morphological traits in mottled spinefoot (*Siganus fuscescens*). The *x*-axis represents the natural logarithm of body length (notochord length before notochord flexion and standard length thereafter, mm), and the *y*-axis represents the natural logarithm of each morphological trait (preanal length, head length, snout length, eye diameter, caudal peduncle depth, body height, head height, and upper jaw length; mm). Points represent individual specimens. Dotted lines indicate the breakpoints in the logarithmic regression.

Segmented regression analysis consistently identified three major breakpoints in multiple body parts, corresponding to NL values of approximately 5 mm, 7–9 mm, and 17–19 mm (Figure 5, Table 1).

#### 3.5.1. First Breakpoint (NL ≈ 5 mm)

The first breakpoint, detected at approximately NL = 4.8–5.3 mm, corresponded to the transition from the preflexion to flexion stage. Prior to this breakpoint (NL ≈ 2.3–5.0 mm), several feeding- and locomotion-related body parts exhibited strong positive allometry relative to NL.

Eye diameter, upper jaw length, snout length, and caudal peduncle depth showed slopes substantially greater than 1.0 (typically b ≈ 1.2–1.5, depending on the trait), indicating disproportionately rapid growth relative to overall body length. In contrast, trunk length and total body length increased more slowly during this phase.

After this breakpoint, the slopes for these feeding-related traits decreased significantly (*p* < 0.05), indicating a clear shift in relative growth pattern.

#### 3.5.2. Second Breakpoint (NL ≈ 7–9 mm)

The second breakpoint was detected at approximately NL = 7.0–9.0 mm, corresponding to the mid-flexion stage. Between the first and second breakpoints (NL ≈ 5–8 mm), most body parts showed reduced allometric slopes, approaching isometric growth (*b* ≈ 1.0).

In this size range, most feeding- and locomotion-related traits exhibited slopes close to unity (*b* ≈ 1.0), indicating near-isometric growth and stabilization of body proportions during this interval.

#### 3.5.3. Third Breakpoint (NL ≈ 17–19 mm)

A third breakpoint was detected at approximately NL = 17–19 mm, corresponding to the transition from the larval to juvenile stage. Beyond this breakpoint (NL > ~18 mm), body depth and caudal peduncle depth again exhibited positive allometry, with slopes increasing to approximately *b* ≈ 1.2–1.4 (*p* < 0.05).

In contrast, head-related measurements, including eye diameter and upper jaw length, showed reduced slopes (*b* < 1.0) after this stage, indicating a relative deceleration of head growth compared with overall body length. Trunk length and standard length increased steadily, contributing to the overall juvenile body form.

### 3.6. Summary of Segmented Growth Patterns

Overall, segmented regression analysis revealed a three-phase pattern of relative growth in *S*. *fuscescens*, characterized by early positive allometry, a mid-larval stabilization phase, and renewed allometric growth during the transition to the juvenile stage.

These statistically identified growth phases and their biological interpretation are summarized in Table 2.

## 4. Discussion

### 4.1. Absolute Growth and Development of S. fuscescens Larvae and Juveniles

The present study revealed a biphasic pattern of absolute growth in notochord length (NL) and standard length (SL) in *S. fuscescens*, characterized by gradual body elongation until approximately 10 dph and a subsequent period of rapid length in-crease during the late larval to early juvenile phase. During the yolk-sac and early larval stages (0–10 dph), NL remained below 5 mm, indicating limited somatic elongation despite the onset of exogenous feeding. Comparable early plateaus in body length have been reported for other marine fishes and are often associated with energetic allocation to organogenesis rather than somatic extension [16,17].

This interpretation aligns with previous studies on digestive tract development in rabbitfishes. Kohno et al. [18] demonstrated that relative gut length (RGL) increases sharply during the first week post-hatching, and similar early digestive differentiation has been documented for herbivorous and omnivorous larvae [11,19]. The modest increase in NL prior to 10 dph therefore likely reflects energetic prioritization toward digestive tract elongation, organ maturation, and initial feeding-related structure development. Such patterns differ from those observed in pelagic carnivores like Pacific bluefin tuna (*Thunnus orientalis*) and greater amberjack (*Seriola dumerili*), in which early elongation of body length is more pronounced [20,21].

After 10 dph, NL increased rapidly to approximately 10–12 mm by 20 dph, and SL exceeded 40 mm by 40 dph. This acceleration coincides with the transition from strong positive allometry in feeding-related traits to near-isometric growth, as revealed by segmented regression in the present study. Such growth shifts correspond to increased locomotor capability and enhanced digestive efficiency, allowing greater energetic allocation toward somatic elongation [22]. The postflexion period thus represents a developmental phase during which larvae gain both structural and functional independence, enabling rapid length growth.

Comparisons with other species provide additional context. In red sea bream (*Pagrus major*), early elongation is stronger, and NL typically reaches 6–7 mm by 8–10 dph under comparable temperatures [23]. Greater amberjack displays even more rapid early elongation, attaining 9–12 mm by 10 dph and transitioning to the juvenile stage sooner [21], whereas Pacific bluefin tuna demonstrate extreme early growth acceleration and surpass 15–20 mm by 10 dph [20]. The slower early elongation of *S. fuscescens* therefore reflects a divergent developmental strategy in which early energy investment emphasizes digestive tract development and feeding-related morphology rather than rapid overall length extension.

It should be noted that, in addition to intrinsic developmental processes, several extrinsic factors not directly controlled in the present study may also have contributed to the observed growth patterns. It should also be noted that early allometric growth patterns in marine fish larvae can be influenced by environmental factors such as rearing temperature, which may modify the timing and magnitude of ontogenetic growth shifts [24]. Stocking density and intra-cohort competition for food can influence growth rates and energy allocation in larval fishes, potentially modulating the timing and magnitude of somatic elongation. Furthermore, changes in feeding regime after 22 dph may have affected digestive efficiency and growth dynamics during later stages. While these factors were maintained within standard hatchery ranges, their individual effects were not experimentally isolated. Therefore, the interpretations presented here should be regarded as functional inferences based on morphological patterns rather than definitive causal relationships.

Finally, the increased variability in SL after 25 dph likely reflects individual differences in feeding success, metabolic efficiency, and early behavioral traits—a pattern also observed in species undergoing settlement transitions [25]. Overall, the absolute growth pattern of *S. fuscescens* underscores a developmental trajectory that prioritizes organogenesis and feeding competence during early ontogeny, followed by rapid somatic elongation once digestive and locomotor capacity has been established. Because no significant among-tank differences in water-quality parameters were detected during the pre-transfer period, the growth patterns and allometric trajectories observed in this study are unlikely to be attributable to systematic environmental heterogeneity among tanks.

### 4.2. Early Allometric Traits of S. fuscescens Larvae and Juveniles in a Comparative Context

The present study revealed three distinctive and interrelated allometric traits in the early development of *S. fuscescens* larvae that clearly differentiate this species from many carnivorous marine fishes.

First, early body deepening was a prominent feature of initial larval development. Between 3 and 8 dph (NL ≈ 2.3–4.3 mm), body depth and head depth increased rapidly from approximately 10–12% to 14–18% of NL, whereas notochord elongation itself remained relatively modest. This pattern contrasts with that observed in pelagic or semi-pelagic larvae such as red sea bream (*Pagrus major*), greater amberjack (*Seriola dumerili*), and Japanese flounder (*Paralichthys olivaceus*), in which early larval growth is typically dominated by elongation of body length with only limited early increases in body depth [21,23,26]. Early body deepening is considered advantageous for posture stabilization and maneuverability [16,27,28], and for predator avoidance [29] in structurally complex coastal and algal-associated habitats, where larvae and early juveniles must maintain orientation and rapidly respond to visual and hydrodynamic cues [27].

Second, the observed temporal shift in body proportions closely corresponded to onto-genetic changes in digestive tract development. Previous studies have shown that *S. fuscescens* larvae undergo a rapid increase in relative gut length (RGL) during the same developmental window (approximately 3–8 dph), followed by continued elongation of the digestive tract at later stages [10,11]. In the present study, early expansion of body depth and head-related dimensions (NL ≈ 2–4 mm) likely reflects enlargement of the body cavity to accommodate rapid gut development during the early larval stage. After NL exceeded approximately 4 mm, body depth ratios stabilized (≈14–17% of NL), while trunk length increased markedly, indicating a shift toward longitudinal growth that supports further elongation of the digestive tract and somatic tissues during later larval and early juvenile stages. This two-phase pattern—early body deepening followed by accelerated body elongation—differs from the more uniform elongation dominated growth reported for many carnivorous species.

Third, early development of feeding-related organs supporting the utilization of small prey items was evident in *S. fuscescens* larvae. Allometric scaling of feeding-related structures has been shown to constrain feeding capacity and prey size selection during early ontogeny in marine fish larvae, thereby linking morphological development directly to feeding performance [30]. Upper jaw length (≈5–7% of NL), head length (≈19–22% of NL), and eye diameter (≈8–10% of NL) were already relatively large immediately after the onset of exogenous feeding and remained high throughout the early larval stage. These proportions are consistent with the well-documented preference of siganid larvae for small rotifers (approximately 60–120 µm), which contrasts with the larger prey sizes (120–180 µm) typically selected by larvae of species such as *P. major* and *S. dumerili* [21,22,26]. Together, these morphological traits indicate that *S. fuscescens* larvae are morphologically specialized for efficient detection and capture of small prey immediately after first feeding, reflecting an ontogenetic strategy distinct from that of pelagic carnivorous fishes and well suited to coastal, al-gal-associated environments.

### 4.3. Developmental Interpretation of Growth Breakpoints Revealed by Segmented Regression

Segmented regression analysis of log–log relationships between body-part dimensions and notochord or standard length revealed three major breakpoints in the relative growth trajectories of *S. fuscescens* larvae, occurring at approximately NL ≈ 5 mm, 7–9 mm, and 17–19 mm (Figure 5, Table 1). Ontogenetic shifts in functional morphology and swimming performance, often occurring as discrete developmental phases, have been reported across a wide range of marine fish larvae [22]. These breakpoints correspond to distinct developmental phases and provide a quantitative framework for interpreting functional and ecological shifts during early ontogeny.

The first breakpoint (NL ≈ 5 mm) coincided with the transition from the preflexion to flexion stage (Figure 3) and was characterized by strong positive allometry in feeding- and locomotion-related traits, including eye diameter, upper jaw length, snout length, and caudal peduncle depth (*b* ≈ 1.2–1.5). Such disproportionate growth indicates rapid acquisition of sensory, prey-capture, and swimming capacities immediately after the onset of exogenous feeding. In many carnivorous marine fishes, such as *Pagrus major*, *Seriola dumerili*, and *Paralichthys olivaceus*, early larval growth is dominated by elongation of body length, while feeding-related structures develop more gradually [20,22]. In contrast, the pronounced early allometry observed in *S. fuscescens* suggests an ontogenetic strategy that prioritizes early functional competence rather than simple body elongation.

The timing of the first breakpoint also corresponds closely with the period of rapid digestive tract development previously reported for siganid larvae. Kohno et al. [18] showed that relative gut length (RGL) in *S. fuscescens* increases sharply during the early larval stage, reaching values exceeding 1.0–1.5 before flexion. The present results indicate that early expansion of body depth and head-related dimensions, together with strong positive allometry of feeding structures, likely reflects coordinated morphological reorganization that supports both internal digestive development and external feeding performance [17,19,22,26,30]. This integrated pattern contrasts with the more uniform growth trajectories observed in pelagic carnivorous species, such as *Seriola lalandi* and other fusiform pelagic predators [31,32], and may be characteristic of herbivorous or omnivorous fishes associated with structurally complex coastal habitats where maneuverability and fine-scale grazing are at a premium [33].

The second breakpoint (NL ≈ 7–9 mm) occurred during the flexion stage and was marked by a transition toward near-isometric growth (*b* ≈ 0.9–1.1) in most body parts. During this phase, previously emphasized traits such as eye diameter, upper jaw length, and head length showed reduced relative growth, indicating stabilization of body proportions. This phase likely represents a period of morphological scaling and adjustment, during which early-specialized structures are integrated into a more balanced body plan. Similar transient phases of proportional stabilization have been reported in other marine fish larvae, although the specific timing and affected traits vary among species [17,22].

The third breakpoint (NL ≈ 17–19 mm) corresponded to the transition from the larval to juvenile stage and was characterized by renewed positive allometry in body depth and caudal peduncle depth (*b* ≈ 1.2–1.4), while head-related traits exhibited relative deceleration (*b* < 1.0). This pattern indicates reorganization of body shape toward the juvenile morphology, with increased emphasis on swimming performance and structural robustness. Comparable late-stage increases in body depth or caudal region development have been reported during metamorphosis in a variety of marine fishes and are commonly associated with shifts in habitat use and locomotor demands [16,22].

Taken together, the three growth breakpoints identified in this study delineate a sequence of functional transitions in *S. fuscescens* larvae: an initial phase of rapid functional acquisition for feeding and locomotion (NL < ~5 mm), a subsequent phase of morphological stabilization and scaling (NL ≈ 5–9 mm), and a final phase of body-shape reorganization associated with juvenile transformation (NL > ~17 mm). This three-phase developmental pattern differs markedly from the predominantly elongation-driven growth observed in many carnivorous marine fishes (Table 3).

This comparative framework highlights fundamental differences in early developmental strategies between *S. fuscescens* and pelagic carnivorous fishes. Whereas pelagic species exhibit rapid early elongation of the body axis to support cruising and pursuit in open-water environments, *S. fuscescens* prioritizes early development of feeding-related and postural structures, resulting in limited initial length increase but strong positive allometry of the head, eyes, jaws, and body depth. These contrasting trajectories indicate that early larval growth is shaped by different functional demands, reflecting divergent ecological strategies between coastal herbivorous/omnivorous fishes and pelagic predators. (Based on [34,35,36,37]).

Accordingly, the functional and ecological interpretations discussed here should be regarded as hypotheses generated from observed morphological patterns, rather than direct demonstrations of causality.

### 4.4. Ecological Implications of Ontogenetic Growth Patterns and Behavior in Natural Habitats

While the present study provides clear implications for hatchery management, the observed ontogenetic patterns of relative growth in *S. fuscescens* may also be informative for interpreting early survival strategies in natural coastal environments, which are characterized by structural complexity, variable light conditions, and heterogeneous prey fields [25,37].

The pronounced early body deepening observed during the early larval stage (NL ≈ 2–4 mm) may be functionally consistent with improved postural stability and maneuverability at low swimming speeds. Increased body and head depth have been associated with enhanced stability and turning performance in complex flow environments in other fishes [33]. However, the present study does not directly evaluate predator–prey interactions or habitat use, and therefore such ecological interpretations should be regarded as hypotheses rather than demonstrated adaptations.

The first growth breakpoint (NL ≈ 5 mm), characterized by strong positive allometry in visual and feeding-related organs, coincides with a period when larvae must efficiently capture small prey. Fluctuations in the availability of suitably sized planktonic prey are common in coastal waters, and enlargement of the eyes and jaws could plausibly enhance prey detection and capture under such conditions, as reported for siganid larvae in previous studies [5,8,25]. Compared with pelagic carnivorous species that forage in relatively homogeneous open-water prey fields, these traits may be functionally relevant in more heterogeneous nearshore environments, although this remains to be tested.

During the second growth phase (NL ≈ 5–9 mm), near-isometric growth of most body parts indicates stabilization of body proportions. This pattern is consistent with a transition from highly specialized early larval morphology toward a more generalized body plan capable of supporting a wider range of locomotor and feeding behaviors, but direct links to habitat shifts were not examined in the present study.

Collectively, the three-phase growth pattern revealed by relative growth and segmented regression analyses describes coordinated changes in morphology that likely influence functional performance through development. While these patterns are consistent with hypotheses about changing ecological demands across ontogeny, the present results should be interpreted primarily as a morphological framework for future experimental and field-based tests.

### 4.5. Integration of Phototactic Behavior and Ontogenetic Changes in Morphology

Visual cues play a central role in prey detection, posture control, and orientation in larval fishes, and visual gradients are known to strongly influence swimming behavior during early development [37].

The ontogenetic changes in relative growth patterns identified in this study provide a morphological framework for interpreting phototactic behavior and associated abnormal behaviors in *S. fuscescens* larvae. During the early larval phase (notochord length [NL] < ~5 mm), pronounced positive allometry in eye diameter, feeding-related structures, and caudal peduncle depth coincides with the period when strong positive phototaxis has been reported in several marine fish larvae at the onset of exogenous feeding [38,39]. This combination of rapid sensory and locomotor development likely increases reliance on visual cues for orientation and prey detection, which under artificial light gradients may elevate the risk of walling and surface-pecking behaviors. Recent hatchery studies have demonstrated that light-dependent swimming behavior can strongly affect larval survival, with inappropriate light environments inducing excessive surface aggregation and walling behavior in marine fish larvae [40].

Experimental studies have further shown that sensorimotor control of posture and orientation in larval fish is tightly regulated by visual gradients, supporting a mechanistic link between visual input and swimming behavior [37,40]. During the intermediate growth phase (NL ≈ 5–9 mm), near-isometric growth of most body parts indicates stabilization of body proportions, which is expected to improve swimming control and reduce dependence on strong light-oriented cues. As larvae approach the late larval to early juvenile stage (NL > ~17 mm), renewed positive allometry in body depth and caudal peduncle depth likely enhances swimming performance and maneuverability, further diminishing the functional importance of phototaxis for orientation and feeding.

However, it should be acknowledged that the proposed relationship between changes in allometric growth patterns and attenuation of phototactic responses is interpretative in nature. The present study did not directly manipulate light environments or quantify behavioral responses across developmental stages; therefore, the links suggested here should be regarded as functional hypotheses rather than demonstrated causal relationships.

Overall, these results suggest that phototactic behavior and its associated abnormal manifestations in *S. fuscescens* are most pronounced during the early functional acquisition phase and decline as morphological stabilization and locomotor capacity increase.

Future studies should explicitly test these hypotheses by manipulating prey size, feeding regime, and light environment according to the allometric thresholds identified here, and by directly quantifying growth, feeding success, and behavior. Such experiments will be necessary to determine whether the morphological patterns described in this study translate into improved hatchery performance.

### 4.6. Implications for Hatchery Management and Feeding Design

The integration of relative growth patterns and behavioral ontogeny has direct implications for hatchery management and feeding strategies in *S. fuscescens* seed production. The early larval phase (NL < ~5 mm), characterized by strong positive allometry in feeding-related organs and heightened sensitivity to visual stimuli, represents a critical window during which larvae are particularly vulnerable to mismatches between morphology, prey size, and light environment. The present results support the use of small rotifers (approximately 60–120 µm) during this phase, a prey size range that is compatible with the relatively large upper jaw length and eye diameter observed immediately after first feeding. Provision of prey exceeding this size range may reduce capture efficiency and promote unnecessary locomotor activity, thereby in-creasing energetic costs.

In addition to prey size, the strong tendency toward phototactic responses during the early larval stage highlights the importance of carefully managing hatchery lighting conditions. Intense overhead illumination can generate steep vertical light gradients that promote surface aggregation and surface-pecking behavior, whereas more homogeneous or mid-depth illumination may help disperse larvae within the water column and reduce abnormal behavior. Comparable adjustments to lighting regimes have been shown to improve survival and reduce behavioral stress in other marine fish larvae [41,42].

During the subsequent stabilization phase (NL ≈ 5–9 mm), when relative growth approaches isometry, larvae appear less dependent on extreme visual cues and more capable of controlled swimming and prey capture. This stage likely represents an optimal window for the gradual increase in prey size and modification of feeding density, as morphological constraints on prey handling are progressively relaxed. Finally, during the late larval to early juvenile phase (NL > ~17 mm), renewed positive allometry in body depth and caudal peduncle depth indicates enhanced swimming performance and structural robustness, supporting the transition to juvenile feeding regimes and more structurally complex rearing environments.

Overall, these findings emphasize that successful fingerling production of *S. fuscescens* requires stage-specific integration of morphology, behavior, and rearing conditions. Rather than applying uniform feeding and lighting protocols throughout larval development, hatchery management strategies should reflect the distinct functional phases revealed by relative growth and segmented regression analyses. Such an approach is expected to reduce early mortality associated with abnormal behaviors and improve overall rearing efficiency for this species.

Although the external development of the intestine was not quantitatively analyzed in the present study, continuous morphological sketches from hatching revealed that intestinal elongation and initial looping began earlier than the stage previously described by Kitajima et al. for postlarval rabbitfish (≥14 dph) [9]. This early expansion of the digestive tract likely contributes to the observed early body deepening, reflecting functional integration between digestive capacity and somatic growth during the onset of exogenous feeding.

## 5. Conclusions

This study demonstrates that early morphological development in *S. fuscescens* follows a distinct three-phase pattern of relative growth, reflecting changing functional requirements during larval and early juvenile stages. Using detailed morphometric measurements and log–log segmented regression analysis, we identified three major developmental phases separated by breakpoints at approximately 5 mm, 7–9 mm, and 17–19 mm in body length.

The earliest larval phase was characterized by strong positive allometry in feeding- and locomotion-related traits, indicating rapid functional acquisition immediately after the onset of exogenous feeding. This was followed by an intermediate phase of near-isometric growth, reflecting stabilization of body proportions, and a later phase marked by renewed positive allometry in body depth and caudal peduncle depth, corresponding to reorganization toward juvenile morphology.

These results highlight an ontogenetic growth strategy in *S. fuscescens* that differs from elongation-dominated patterns reported in many carnivorous marine fishes. The application of segmented regression provided an effective framework for detecting functionally meaningful growth transitions and offers a useful approach for comparative developmental studies. From an applied perspective, recognizing these stage-specific growth patterns can contribute to the optimization of feeding and rearing strategies in rabbitfish aquaculture. Future experimental studies manipulating stocking density, feeding regime, or light environment are required to directly test the functional implications inferred from the present morphological patterns.

It should be noted that digestive tract function and feeding performance were not directly measured in this study, and thus functional interpretations based on allometric patterns remain inferential. Future studies combining morphometrics with experimental manipulation of diet and light environment will be necessary to test these hypotheses.

It should be emphasized that the present study provides a morphological and statistical framework for understanding early ontogenetic growth in *S. fuscescens*. Direct experimental validation of functional performance, digestive capacity, and behavioral responses will be necessary in future studies to test the hypotheses inferred from the observed allometric patterns.

## Figures and Tables

**Table 1:** Estimated breakpoints and allometric slopes for each morphometric trait based on log–log segmented regression. For each body part, the first (BP1) and second (BP2) breakpoints (mm) indicate transitions between three growth phases. Slopes represent allometric exponents (*b*) for each phase. 95% confidence intervals (CI) are provided for breakpoint estimates and slope coefficients, allowing assessment of the uncertainty associated with each parameter.

Body Part	BP1 (mm)	BP1 95% CI	BP2 (mm)	BP2 95% CI	Slope 1	Slope 1 95% CI	Slope 2	Slope 2 95% CI	Slope 3	Slope 3 95% CI
Pre-anal length (mm)	5.05	4.31–5.79	9.57	8.69–10.45	0.38	0.14–0.62	0.63	0.59–0.67	0.44	0.42–0.46
Snout length (mm)	5.10	4.71–5.49	7.67	6.94–8.40	0.04	−0.02–0.10	0.19	0.17–0.21	0.13	0.11–0.15
Caudal peduncle depth (mm)	5.07	4.62–5.52	7.44	7.19–7.69	0.07	0.01–0.13	0.18	0.16–0.20	0.03	0.03–0.03
Head depth (mm)	4.97	4.30–5.64	7.58	7.07–8.09	0.24	0.08–0.40	0.43	0.37–0.49	0.24	0.22–0.26
Total length (mm)	7.10	6.53–7.67	10.33	9.27–11.39	1.08	1.04–1.12	1.38	1.28–1.48	1.19	1.19–1.19
Head length (mm)	5.09	4.38–5.80	7.52	6.83–8.21	0.20	0.00–0.40	0.44	0.36–0.52	0.22	0.12–0.32
Eye diameter (mm)	5.16	4.12–6.20	7.22	6.69–7.75	0.11	0.05–0.17	0.17	0.13–0.21	0.09	0.09–0.09
Body depth (mm)	5.09	4.01–6.17	8.17	7.64–8.70	0.29	0.11–0.47	0.42	0.38–0.46	0.22	0.20–0.24
Upper jaw length (mm)	5.70	4.43–6.97	8.40	7.89–8.91	0.18	0.08–0.28	0.29	0.23–0.35	0.02	0.00–0.04

Note: Slopes represent allometric coefficients (*b*) estimated from ln–ln segmented regression between each morphological trait and body length. BP indicates the estimated breakpoint (body length at which a change in allometric slope occurs) identified by segmented regression analysis.

**Table 2:** Summary of statistically identified breakpoints and their biological interpretation in *S. fuscescens*.

Developmental Phase	Body Length Range (NL/SL)	Statistically Detected Breakpoint(s)	Dominant Allometric Pattern	Major Traits Involved	Biological/Functional Interpretation
Phase I: Early larval phase	NL < ~5 mm	First breakpoint (≈4.8–5.3 mm)	Strong positive allometry (b > 1)	Eye diameter, upper jaw length, snout length, caudal peduncle depth	Rapid acquisition of visual, feeding, and initial swimming functions immediately after first feeding
Phase II: Mid-larval phase	NL ≈ 5–9 mm	Second breakpoint (≈7–9 mm)	Near-isometric growth (b ≈ 1)	Most body parts	Stabilization and scaling of body proportions following early functional specialization
Phase III: Late larval–early juvenile phase	NL > ~17 mm (SL thereafter)	Third breakpoint (≈17–19 mm)	Renewed positive allometry in trunk-related traits	Body depth, caudal peduncle depth	Reorganization of body shape toward juvenile morphology and enhanced swimming performance

**Table 3:** Comparative framework between *S. fuscescens* and pelagic carnivorous fishes during early ontogeny.

Feature	*S. fuscescens* (Rabbitfish, Coastal Herbivore/Omnivore)	Pelagic Carnivorous Fishes (e.g., *Thunnus orientalis*, *Seriola dumerili*, *Euthynnus affinis*)
Adult habitat	Coastal, reef- and algal-associated environments	Open ocean, pelagic waters
Early larval habitat (inferred)	Coastal and nearshore waters with complex visual backgrounds	Pelagic, relatively homogeneous light and prey fields
Early growth strategy	Limited early somatic elongation; emphasis on sensory, feeding, and postural structures	Rapid elongation of body axis for early cruising and pursuit
Body length increase during first feeding	Slow; NL remains < ~5 mm during 0–10 dph	Fast; many species reach >10 mm within 7–10 dph
Early allometry of feeding-related traits	Strong positive allometry of eye, jaw, snout, and head dimensions	Moderate allometry; body axis elongation dominates
Early allometry of body depth	Positive early allometry, indicating early body deepening	Often near-isometric or delayed deepening
Functional emphasis in early larvae	Visual prey detection, precise capture, and maneuverability at low speeds	Swimming endurance, pursuit speed, and early piscivory
Timing of morphological stabilization	Intermediate phase (NL ≈ 5–9 mm) with near-isometric growth	Often later, after rapid elongation phase
Ontogenetic trajectory	Three-phase growth with early specialization, stabilization, then juvenile reorganization	Strong early elongation followed by later body deepening and fin development
Ecological implication (inferred)	Suited to variable, complex nearshore prey environments	Suited to open-water prey fields and active pursuit

## Data Availability

The data are available from the corresponding author upon reasonable request.

## Supplementary Materials

The following supporting information can be downloaded at: https://www.mdpi.com/article/10.3390/ani16050777/s1, Table S1: Results of linear models testing among-tank differences in water-quality parameters during the pre-transfer period (0–15 dph). Linear models were fitted with days post-hatching (dph) and time of day (AM/PM) as covariates and tank as a factor. Statistical analyses were restricted to the pre-transfer period (0–15 dph), because larvae were transferred and pooled among tanks after 16 dph.

## Abbreviations

BL	Body length
NL	Notochord length
SL	Standard length
TL	Total length
PAL	Pre anal length
HL	Head length
SnL	Snout length
BH	Body height
HH	Head height
CPD	Caudal peduncle height
UJL	Upper jaw length
ED	Eye diameter
BP	Break Point
