# Supplementary material for: Allometric Growth of Feeding and Locomotor Structures During Early Ontogeny of Rabbitfish (Siganus fuscescens)
Source: Animals (Basel). 2026 Mar 2;16(5):777. doi: 10.3390/ani16050777 (PMC12984535; doi:10.3390/ani16050777)
Supplement: Supplementary file 1 [file animals-16-00777-s001.zip › animals-4091076-supplementary.pdf]

Supplementary Materials

Table S1. Results of linear models testing among-tank differences in water-quality parameters during the pre-transfer period (0–15 dph).

Variable	Effect	df	F value	p value
Temperature (° C)	dph	1,118	0.46	0.498
	Time (AM/PM)	1,118	3.68	0.057
	Tank	4,118	1.68	0.159
Dissolved oxygen (%)	dph	1,123	0.99	0.321
	Time (AM/PM)	1,123	3.83	0.053
	Tank	4,123	0.50	0.738
Salinity	dph	1,123	23.53	<0.001
	Time (AM/PM)	1,123	4.84	0.030
	Tank	4,123	1.17	0.325
pH	dph	1,123	63.59	<0.001
	Time (AM/PM)	1,123	1.61	0.208
	Tank	4,123	0.59	0.673

Linear models were fitted with days post-hatching (dph) and time of day (AM/PM) as covariates and tank as a factor. Statistical analyses were restricted to the pre-transfer period (0–15 dph), because larvae were transferred and pooled among tanks after 16 dph.
